# Transcriptional Regulation of the Type VI Secretion System 1 Genes by Quorum Sensing and ToxR in *Vibrio parahaemolyticus*

**DOI:** 10.3389/fmicb.2017.02005

**Published:** 2017-10-16

**Authors:** Yiquan Zhang, He Gao, George Osei-Adjei, Ying Zhang, Wenhui Yang, Huiying Yang, Zhe Yin, Xinxiang Huang, Dongsheng Zhou

**Affiliations:** ^1^School of Medicine, Jiangsu University, Zhenjiang, China; ^2^State Key Laboratory for Infectious Disease Prevention and Control, National Institute for Communicable Disease Control and Prevention, Chinese Centre for Disease Control and Prevention, Beijing, China; ^3^Department of Biosafety, State Key Laboratory of Pathogen and Biosecurity, Beijing Institute of Microbiology and Epidemiology, Beijing, China

**Keywords:** *Vibrio parahaemolyticus*, T6SS1, quorum sensing, AphA, OpaR, ToxR

## Abstract

*Vibrio parahaemolyticus*, the leading cause of seafood-associated gastroenteritis, harbors two separate T6SSs on chromosomes 1 and 2, i.e., T6SS1 (VP1386-1420) and T6SS2 (VPA1025-1046). T6SS1 contains at least 7 putative operons: VP1386-1387, VP1388-1390, VP1392-1391, VP1393-1406, VP1400-1406, VP1409-1407, and VP1410-1420. *V. parahaemolyticus* AphA and OpaR are the two master regulators of quorum sensing (QS) system that are highly expressed at low cell density and high cell density, respectively. ToxR is a membrane-bound virulence regulatory protein conserved across the *Vibrio* family. In the present work, we show that ToxR coordinates with AphA and OpaR to repress T6SS1 expression in *V. parahaemolyticus*. OpaR binds to the promoters of VP1388-1390, VP1400-1406, and VP1409-1407 to repress their transcription, but it appears to negatively regulate VP1393-1406 transcription in an indirect manner. By contrast, AphA negatively regulated the above four T6SS1 operons in an indirect manner. In addition, ToxR binds to the promoters of VP1400-1406 and VP1409-1407 to inhibit their transcription, but it presents an indirect interaction with VP1388-1390 and VP1393-1406 promoters. Notably, the expression of ToxR also manifested in a QS-dependent manner and the highest expression occurred at LCD. Meanwhile, the highest expression of T6SS1 occurred at an OD_600_ value of 0.6 to 0.8 due to the tight regulation of ToxR and QS, suggesting T6SS1 functions only during the mid-logarithmic growth phase. These observations provide significant insight into the molecular mechanism of T6SS1 gene regulation by QS and ToxR in *V. parahaemolyticus*.

## Introduction

*Vibrio parahaemolyticus* is a Gram-negative halophilic bacterium that is mostly disseminated in marine and estuarine surroundings (Broberg et al., 2011; Lovell, 2017). Virulent *V. parahaemolyticus* strains usually cause human acute gastroenteritis after consumption of raw or poorly cooked seafood (Broberg et al., 2011). In rare cases, *V. parahaemolyticus* also causes skin infection or septicaemia if the bacterium enters into an open wound (Broberg et al., 2011). *V. parahaemolyticus* strains expresses a number of different virulence factors including thermostable direct hemolysin (*tdh*), TDH related hemolysin (*trh*), two type VI secretion systems (T6SS1 and T6SS2) as well as two type III secretion systems (T3SS1 and T3SS2) (Broberg et al., 2011). These factors play important roles in the strains surviving in the environment and causing human disease.

The T6SS is a newly described mechanism in Gram-negative bacteria that transports protein effectors into a diversity of recipient cells (Records, 2011). *V. parahaemolyticus* strain RIMD2210633 harbors two different T6SSs on chromosomes 1 and 2, named as T6SS1 (VP1386-1420) and T6SS2 (VPA1025-1046), respectively (Makino et al., 2003; Li et al., 2017). The T6SS1 locus is composed of 34 consecutive genes forming at least 7 putative operons, i.e., VP1386-1387, VP1388-1390, VP1392-1391, VP1393-1406, VP1400-1406, VP1409-1407, and VP1410-1420, while the T6SS2 locus contains 22 genes in 3 putative operons, i.e., VPA1027-1025, VPA1043-1028, and VPA1044-1046 (Makino et al., 2003; Ma et al., 2012) (**Supplementary Figure S1**). The T6SS1 has been thought that predominantly present in clinical isolates of *V. parahaemolyticus*, but a recent study demonstrated that all of the environmental acute hepatopancreatic necrosis disease (AHPND) strains, but none of the non-AHPND strains, contain the T6SS1 (Li et al., 2017). By contrast, the T6SS2 is found in all tested strains of *V. parahaemolyticus* including both environmental and clinical strains (Yu et al., 2012). The T6SS1 is most active under high salt conditions (3% sodium chloride) at warm temperatures (30°C), while the T6SS2 is only active under low salt conditions (such as in Luria-Bertani broth) (Salomon et al., 2013). Thus, the T6SS1 would enhance environmental fitness of *V. parahaemolyticus* in sea water, while the T6SS2 functions when the bacterium is inside a marine animal (Salomon et al., 2013).

Quorum sensing (QS) is the process of cell-cell signaling that mediates communal behavior and gene regulation in response to the presence of chemical signals known as autoinducers (AIs) (Waters and Bassler, 2005; Defoirdt et al., 2008; Srivastava and Waters, 2012). QS was first discovered in the marine bacterium *V. fischeri* but later found to be present in many other bacteria (Defoirdt et al., 2008). QS is involved in controlling a variety of cellular pathways, including virulence factor production, biofilm formation, drug resistance, and motility (Defoirdt et al., 2008). QS regulates target gene expression via the downstream master QS regulators (Ng and Bassler, 2009). AphA and OpaR are the two master regulators of QS in *V. parahaemolyticus* (Sun et al., 2012; Zhang et al., 2012). The highest expression level of AphA occurred at low cell density (LCD) or low concentrations of AIs, whereas that of OpaR appeared at high cell density (HCD) or high concentrations of AIs, thus AphA and OpaR function at LCD and HCD, respectively (Sun et al., 2012; Zhang et al., 2012). AphA and OpaR individually or combined, regulate hundreds of target genes during QS signal transduction between LCD and HCD (Gode-Potratz and McCarter, 2011; van Kessel et al., 2013). The asymmetrical cell density-dependent production of AphA and OpaR establish a precise temporal pattern of gene expression in *V. parahaemolyticus*.

ToxR is a membrane-bound regulatory protein that plays roles in the virulence and fitness in *Vibrio* species (Childers and Klose, 2007). In *V. cholerae*, ToxR acts with TcpP to induce *toxT* transcription, ToxT then induces expression of the two major virulence determinants, i.e., cholera toxin and the toxin co-regulated pilus (DiRita et al., 1991). ToxR alone can also directly activate the *ctxAB* promoter in the presence of bile, suggesting a direct role of ToxR in *in vivo* cholera toxin expression (Hung and Mekalanos, 2005). ToxR alone also has regulatory activity on the transcription *ompU* and *ompT*, resulting in activation of OmpU and repression of OmpT, which are required for *V. cholerae* to survive in stress conditions (Provenzano and Klose, 2000; Merrell et al., 2001; Mathur and Waldor, 2004; Goss et al., 2013). Goss et al. (2013) previously defined the sequence TNAAA-N5-TNAAA as the ToxR-binding motif by analyzing the ToxR-footprinted region of the *toxT*, *ompU*, *ompT*, and *ctxA* promoters (Goss et al., 2013). ToxR in *V. parahaemolyticus* shares high similarities with *V. cholerae* ToxR, and thus they may share a similar function, i.e., regulate the expression of major virulence determinants (Lin et al., 1993). Previous studies showed that *V. parahaemolyticus* ToxR activates the expression of TDH, T3SS2 and OmpU, while it represses T3SS1 expression (Lin et al., 1993; Whitaker et al., 2012; Hubbard et al., 2016). A recent shudy showed that ToxR represses T3SS1 genes transcription via direct activation of CalR, which acts as a direct repressor of T3SS1 genes (Osei-Adjei et al., 2017).

Although the detailed mechanisms have not been fully elucidated, QS and ToxR-dependent expression of T6SS has been established in *V. parahaemolyticus* (Gode-Potratz and McCarter, 2011; Ma et al., 2012; Wang et al., 2013; Salomon et al., 2014). OpaR represses T6SS1 but activates T6SS2 at HCD, whereas AphA activates T6SS1 but represses T6SS2 at LCD (Gode-Potratz and McCarter, 2011; Ma et al., 2012; Wang et al., 2013). Deletion of *toxR* has a mild negative effect on *hcp1* (a T6SS1 gene) expression compared to the parental strain under non-optimal inducing conditions (Salomon et al., 2014). In this article, we show that the virulence regulator ToxR coordinates with QS regulator AphA and OpaR to repress T6SS1 in *V. parahaemolyticus* strain RIMD2210633. The highest expression of T6SS1 genes occurred at an OD_600_ value of 0.6 to 0.8 due to the tight regulation of ToxR and QS, suggesting T6SS1 functions only during the mid-logarithmic growth phase in *V. parahaemolyticus*.

## Materials and Methods

### Bacterial Strains

The *V. parahaemolyticus* strain RIMD2210633 was used as the wild type (WT) in this study (Makino et al., 2003). The non-polar *aphA* and *opaR* single-gene deletion mutants derived from WT (designated as Δ*aphA* and Δ*opaR*, respectively) were described in our previous studies (Sun et al., 2012; Zhang et al., 2012) (shown also in **Supplementary Figure S2**). For construction of *toxR* deletion mutant (Δ*toxR*), the 428 and 455 bp DNA regions upstream and downstream of *toxR* were amplified by PCR, purified, and used as the templates to create an 879 bp deletion construct that was subsequently inserted between the *Pst* I and *Sph* I sites of pDS132 (Philippe et al., 2004). After being verified by DNA sequencing, the recombinant vector was transformed into *Escherichia coli* S17-bbbpir, and then transferred into WT by conjugation (Philippe et al., 2004). The mutant strain was selected using resistance to 10% sucrose and sensitivity to 5 μg/ml chloramphenicol, and further verified by PCR. All the primers used were listed in **Table 1**.

**Table 1:** Oligonucleotide primers used in this study.

Target	Primers (forward/reverse, 5′–3′)	Reference
**Construction of mutants**		
*aphA*	GTGACTGCAGCGCAGCAAATAACCAGAC/CCAATCACTTCAAGTTCTGTTGTCTTCAATCCAAATGGTC	Sun et al., 2012
	GACCATTTGGATTGAAGACAACAGAACTTGAAGTGATTGG/GTGAGCATGCGTTTTCGTGACCGCTGTG	
	GTGACTGCAGCGCAGCAAATAACCAGAC/GTGAGCATGCGTTTTCGTGACCGCTGTG	
*opaR*	GTGACTGCAGACTGCCTTGGTAACGCTCTG/GTTCGTGTTCAAATCTGAGCTATCCATTTTCCTTGCCATTTG	Zhang et al., 2012
	CAAATGGCAAGGAAAATGGATAGCTCAGATTTGAACACGAAC/GTGAGCATGCATGGGCTGCATCAGGTCG	
	GTGACTGCAGACTGCCTTGGTAACGCTCTG/GTGAGCATGCATGGGCTGCATCAGGTCG	
*toxR*	GTGACTGCAGAAACGCAATTTGTCTGATG/ATCTTCATGCTGGCCTCCTTTAGTTCTTCTTAGATGGATGATG	This study
	CATCATCCATCTAAGAAGAACTAAAGGAGGCCAGCATGAAGAT/GTGAGCATGCAATTCGGCGGCTTTGTTC	
	GTGACTGCAGAAACGCAATTTGTCTGATG/GTGAGCATGCAATTCGGCGGCTTTGTTC	
**Construction of complemented mutants**		
*aphA*	GATTCTAGAAGGAGGAATTCACCATGTCATTACCACACGTAATC/GACAAGCTTTTAACCAATCACTTCAAGTTC	Sun et al., 2012
*opaR*	GATTCTAGAAGGAGGAATTCACCATGGACTCAATTGCAAAGAG/GACAAGCTTTTAGTGTTCGCGATTGTAG	Zhang et al., 2012
*toxR*	GATTCTAGAAGGAGGAATTCACCATGACTAACATCGGCACCAA/GACAAGCTTTTATTTGCAGATGTCTGTTGG	This study
**Protein expression**		
*aphA*	AGCGGGATCCATGTCATTACCACACGTAATC/AGCGAAGCTTTTAACCAATCACTTCAAGTTC	Sun et al., 2012
*opaR*	AGCGGGATCCATGGACTCAATTGCAAAGAG/AGCGAAGCTTTTAGTGTTCGCGATTGTAG	Zhang et al., 2012
*toxR*	AGCGGGATCCATGACTAACATCGGCACCAA/AGCGAAGCTTTTAAGGATTCACAGCAGAAG	This study
**qRT-PCR**		
VP1388	CGTCCTTACACCTGATGAG/TGTCGAATAGCCGTTAG	This study
*hcp1*	GGTCAACCTACTGGTCAACG/TAGTGCTCTTGCTTGCCTTG	This study
VP1400	GTATTAGACACGTTGCCATC/ATCTGCTTGCCTCATTCG	This study
VP1409	TTCTGTGCTCGACTTGTG/TTCAGTGTACTCAACCATCC	This study
**Primer extension**		
VP1388	/ GATAGCTCGTTGGAGGAAAG	This study
*hcp1*	/ GAGTTTCACCGTTGATAGAC	This study
VP1400	/ ACACTTTGCACTCTTTTATGG	This study
VP1409	/ TTAGACAATAAAAAGCCGA	This study
**LacZ fusion**		
VP1388	AAAGTCGACCAATGGTGAATATGCCGTG/AAGGTACCGATAGCTCGTTGGAGGAAAG	This study
*hcp1*	GCGCGTCGACGCTATCGGGTGTAGACGCTG/GCGCGAATTCGAGTTTCACCGTTGATAGAC	This study
VP1400	GAGGTCGACTTTTGCCGAAGAAATAC/TATAGAATTCGCCAATCTTTGCTCGTTCAG	This study
VP1409	ATATGTCGACAACACATGGCATAAATGAGTCC/CCCGAATTCTCTTCTTGTGAAGTCGCTGAA	This study
**EMSA**		
VP1388	CAATGGTGAATATGCCGTG/GATAGCTCGTTGGAGGAAAG	This study
*hcp1*	GCTATCGGGTGTAGACGCTG/GAGTTTCACCGTTGATAGAC	This study
VP1400	TTTTGCCGAAGAAATAC/GCCAATCTTTGCTCGTTCAG	This study
VP1409	AACACATGGCATAAATGAGTCC/TCTTCTTGTGAAGTCGCTGAA	This study
**DNase I footprinting**		
VP1388	TTGAATTTGTTAACTTCTGCC/ATTCTCCACAACCAACATCG	This study
VP1400	GCGTGCTAATCATTCTTTG/ACACTTTGCACTCTTTTATGG	This study
	GCGTGCTAATCATTCTTTG/ACACTTTGCACTCTTTTATGG	This study
VP1409	ATTAGGAACAAAGATCACACA/CGCTTGGATTACTTTCATCG	This study
	AAGTTTATTAAAATGTTC/GCCTGCTATGCCAAATGA	This study
	GAACAAAGATCACACAAAATGGA/CGCTTGGATTACTTTCATCG	This study

For complementation of the mutants (Sun et al., 2014), a PCR-generated DNA fragment containing the coding region together with an upstream synthetic ribosome binding Shine-Dalgarno (SD) sequence (AGGAGG) (Shine and Dalgarno, 1974) for each deleted gene was inserted between the *Xba* I and *Hind* III sites of the pBAD33 (Guzman et al., 1995) vector harboring an arabinose P_BAD_ promoter and a chloramphenicol resistance gene. After being verified by DNA sequencing, the recombinant plasmid for each gene was transformed into the corresponding mutant, yielding the complemented mutant strain Δ*aphA/*pBAD33-*aphA*, Δ*opaR/*pBAD33-*opaR*, *or*Δ*toxR/*pBAD33-*toxR*. For controls, the empty vector pBAD33 was also transformed into WT and each mutant to counteract the effects of arabinose and chloramphenicol on bacterial growth and physiology.

### Bacterial Growth

*Vibrio parahaemolyticus* strains were cultured in Difco marine broth 2216 (BD Biosciences) at 30°C with shaking at 200 rpm. The glyceric stock of bacterial cells were inoculated into 5 ml of M broth and incubated overnight for at least 12 h. The overnight cell cultures were diluted 1:50 into 15 ml of fresh M broth, and grown to reach at OD_600_≈1.0, and then diluted 1:1000 into 15 ml of marine broth for the third-round growth, and were harvested at required cell densities. The culture medium was supplemented with 50 μg/ml gentamicin, 5 μg/ml chloramphenicol, or 0.1% arabinose where necessary. *V. parahaemolyticus* is a biosafety level 2 (BSL-2) pathogen, and thus all the experimental operations involving in live bacteria were done in the BSL-2 lab.

### RNA Isolation and Quantitative Real-Time PCR (qRT-PCR)

Total bacterial RNAs were extracted using the TRIzol Reagent (Invitrogen, United States). The contaminated genome DNA in the total RNAs was removed by using the Ambion’s DNA-free^TM^ Kit according to the manufacturer’s instructions. cDNAs were generated by using 3 ∼ 8 μg of total RNAs and 3 μg of random hexamer primers. The SYBR Green qRT-PCR assay was performed and analyzed as previously described (Gao et al., 2011). The relative mRNA levels were determined based on the standard curve of 16S rRNA (reference gene) expression for each RNA preparation.

### Primer Extension Assay

For the primer extension assay (Sun et al., 2012; Zhang et al., 2012), an oligonucleotide primer complementary to a portion of the RNA transcript of each indicated gene was employed to synthesize cDNAs from total RNA templates. Approximately 10 μg of total RNAs were annealed with 1 pmol of 5′- ^32^P-end labeled reverse oligonucleotide primer to generate cDNAs using a Primer Extension System (Promega, United States). The same labeled primer was used for sequencing with the AccuPower and Top DNA Sequencing Kit (Bioneer, South Korea). The primer extension products and sequencing materials were concentrated and analyzed in an 8 M urea-6% polyacrylamide gel electrophoresis, and the results were detected by autoradiography with the Fuji Medical X-ray film (Fuji Photo Film Co., Ltd., Japan).

### LacZ Fusion and β-Galactosidase Assay

For the LacZ fusion and β-galactosidase assay (Sun et al., 2012, 2014), the promoter DNA region of each indicated gene was amplified by PCR with *ExTaq*^TM^ DNA polymerase (Takara, Japan) using the genomic DNA as the template. PCR amplicons were cloned into the corresponding restriction endonuclease sites of pHRP309 plasmid harboring a promoterless *lacZ* reporter gene and a gentamicin resistance gene (Parales and Harwood, 1993). After being verified by DNA sequencing, the recombinant plasmid was transferred into WT and mutant strains, respectively. An empty pHRP309 plasmid was also introduced into each strain and tested as the negative control. The *V. parahaemolyticus* strains transformed with recombinant or empty pHRP309 plasmids were cultivated as above to measure the β-galactosidase activity in cellular extracts using a β-Galactosidase Enzyme Assay System (Promega, United States) according to the manufacturer’s instructions.

### Preparation of 6× His-Tagged Proteins

The entire coding region of *aphA*, *opaR*, and the truncated *toxR* (1–528 bp, a.a.1–176) of the strain RIMD 2210633 were amplified, purified, and cloned into plasmid pET28a (Novagen, United States), respectively. The recombinant plasmid encoding His-tagged protein was transformed into *E. coli* BL21bbbDE3 cells for protein expression (Kleberjanke and Becker, 2000). Expression and purification of His-AphA and His-OpaR have been described previously (Sun et al., 2012; Zhang et al., 2012), while His-ToxR was the same as that of His-AphA.

### Electrophoretic Mobility Shift Assay (EMSA)

For EMSA (Sun et al., 2012; Zhang et al., 2012), the 5′-ends of the promoter-proximal DNA region of each indicated gene were labeled using [γ-^32^P] ATP and T4 polynucleotide kinase. DNA binding was performed in a 10 μl reaction volume containing binding buffer (1 mM MgCl_2_, 0.5 mM EDTA, 0.5 mM DTT, 50 mM NaCl, 10 mM Tris-HCl/pH 7.5, and 10 mg/ml salmon sperm DNA), labeled DNA (1000–2000 CPM/μl), and increasing amounts of His-tagged protein. Three controls were included in each EMSA experiment: (1) cold probe as specific DNA competitor (the same promoter-proximal DNA region unlabeled), (2) negative probe as non-specific DNA competitor (the unlabeled coding region of the 16S rRNA gene), and (3) non-specific protein competitor (rabbit anti-F1-protein polyclonal antibodies). After incubation at room temperature for 30 min, the products were loaded onto a native 4% (w/v) polyacrylamide gel, and electrophoresed in 0.5× TBE buffer for about 50 min at 200 V. Radioactive species were detected by autoradiography after exposure to Fuji Medical X-ray film at -20°C.

### DNase I Footprinting

For DNase I footprinting (Sun et al., 2012; Zhang et al., 2012), the target promoter DNA regions with a single ^32^P-labeled end were PCR amplified with either sense or antisense primer being end-labeled. The PCR products were purified using the QiaQuick columns (Qiagen, Germany). Increasing amounts of His-tagged protein were incubated with the purified, labeled DNA fragment (2–5 pmol) for 30 min at room temperature, in a final 10 μl reaction volume containing the binding buffer used in EMSA. Before DNA digestion, 10 μl of Ca^2+^/Mg^2+^ solution (5 mM CaCl_2_ and 10 mM MgCl_2_) was added, followed by incubation for 1 min at room temperature. The optimized RQ1 RNase-Free DNase I (Promega, United States) was then added to the reaction mixture, and the mixture was incubated at room temperature for 40–90 s. The reaction was quenched by adding 9 μl of stop solution (200 mM NaCl, 30 mM EDTA, and 1% SDS), followed by incubation for 1 min at room temperature. The partially digested DNA samples were extracted with phenol/chloroform, precipitated with ethanol, and analyzed in 6% polyacrylamide/8 M urea gel. Protected regions were identified by comparison with the sequence ladders. The templates for DNA sequencing were the same as the DNA fragments for DNase I footprinting assay. Radioactive species were detected by autoradiography after exposure to Fuji Medical X-ray film at -20°C.

### Experimental Replicates and Statistical Methods

The LacZ fusion assay and qRT-PCR were performed with at least three independent bacterial cultures and the values were expressed as mean ± standard deviation. Paired Student’s *t*-test was used to calculate statistically significant differences, *p* < 0.01 was considered to indicate statistical significance. The presented data of primer extension, EMSA, and DNase I footprinting assays were done with at least two independent biological replicates.

## Results

### Predicted AphA/OpaR/ToxR Box-Like Sequences within T6SS1 Locus

VP1393-1406 and VP1392-1391 (also VP1409-1407 and VP1410-1420) are adjacent but are transcribed in the opposite direction and they share the same intergenic DNA region (**Supplementary Figure S1**). Thus, the 400 bp upstream regions of VP1386-1387, VP1388-1390, VP1393-1406, VP1400-1406, and VP1409-1407 in the T6SS1 gene cluster were retrieved from the genome sequence of RIMD 2210633 with the ‘*retrieve-sequence*’^1^. Subsequently, the DNA binding boxes of AphA (Sun et al., 2012), OpaR (Zhang et al., 2012), and ToxR (Goss et al., 2013) were used to statistically predict the presence of AphA/OpaR/ToxR box-like sequences within the above target upstream regions by using the *matrix-scan* toolaaa. The analysis generated the weight scores for each target upstream region. The higher score values represented the higher probability of regulatory protein and upstream region association. When the weight score of six was taken as the cutoff value, the OpaR box-like sequences were found for VP1388-1390, VP1400-1406 and VP1409-1407, while the ToxR box-like sequences were found for VP1386-1387 and VP1409-1407 (**Table 2**). However, the AphA box-like sequences were not found in all of the upstream regions tested. Thus, the first genes of VP1388-1390, VP1393-1406, VP1400-1406 and VP1409-1407 were selected for the following gene regulation studies.

**Table 2:** Predicted AphA/OpaR/ToxR box-like sequences within upstream DNA regions.

Operon	First gene	AphA box-like sequence			OpaR box-like sequence			ToxR box-like sequence		
		Position^&^	Sequence	Score	Position^&^	Sequence	Score	Position^&^	Sequence	Score
VP1386-1387	VP1386	NA	NA	NA	NA	NA	NA	R	GCAAAAAAACTAAAA	7.1
VP1388-1390	VP1388	NA	NA	NA	D	TGTTATGAATTAAATTAGTA	7.5	NA	NA	NA
VP1393-1406	*hcp1*	R	TAAATCACCAAATCGCTTAT	1.84	D	ATTGAATCAATTTATTATTA	4.8	NA	NA	NA
VP1400-1406	VP1400	NA	NA	NA	D	TAATTATAACTAAAATAATA	6.82	NA	NA	NA
VP1409-1407	VP1409	R	CTATGCCAAATGACATGCAA	0.39	D	AATTGATATTAATATTAGGA	6.6	D	TAAAATCAGAAAAAA	6.8
								R	TAAATCTAATTAAAA	6.9

*&, ‘D’ indicates the direct sequence, while ‘R’ the reverse one*.

### Cell Density-Dependent Transcription of T6SS1 Genes

**FIGURE 1:**
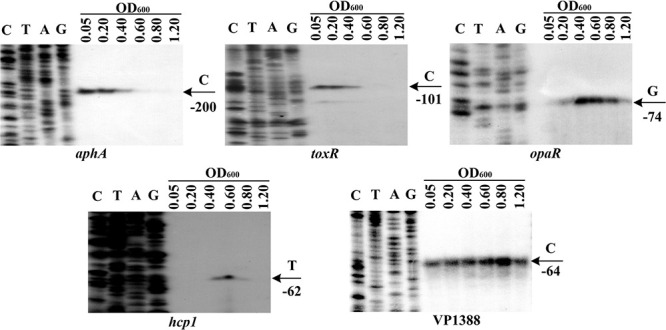
Cell density-dependent expression of target genes. Lanes C, T, A, and G represented Sanger sequencing reactions. The WT cells were harvested at various OD_600_ values. An oligonucleotide primer was designed to be complementary to the RNA transcript of each gene tested. The primer extension products were analyzed with an 8 M urea-6% acrylamide sequencing gel. The transcription start sites were indicated by arrows with nucleotides and positions.

The mRNA levels of *aphA*, *opaR*, *toxR*, VP1388 and VP1393 (*hcp1*) were measured in WT grown at different cell densities by the primer extension assay (**Figure 1**). The *aphA* and *toxR* mRNA levels decreased considerably with the increasing of cell density, and the highest transcription appeared at an OD_600_ value of 0.05 to 0.2; when the OD_600_ value was higher than 0.4, the mRNAs of both *aphA* and *toxR* were undetectable. On the contrary, the *opaR* mRNA level was increased but then reduced with the increasing of cell density, and the highest transcription occurred at an OD_600_ value of 0.4 to 0.6; when the OD_600_ value was lower than 0.2, the *opaR* mRNA was undetectable. The highest transcription level of *hcp1* emerged at an OD_600_ value of 0.6; when the OD_600_ value was lower than 0.4 or higher than 0.8, the *hcp1* mRNA was undetectable. However, the VP1388 mRNAs were detected at all cell densities, and the highest transcription occurred at an OD_600_ value of 0.8. Thus, the bacterial cells were harvested at an OD_600_ value of about 0.15 and 0.4–0.6 for characterizing AphA/ToxR- and OpaR-mediated gene regulation, respectively. In addition, the cell density-dependent transcription of VP1388 and *hcp1* suggests T6SS1 expression would be under control of QS.

### Negative Regulation of T6SS1 by AphA

**FIGURE 2:**
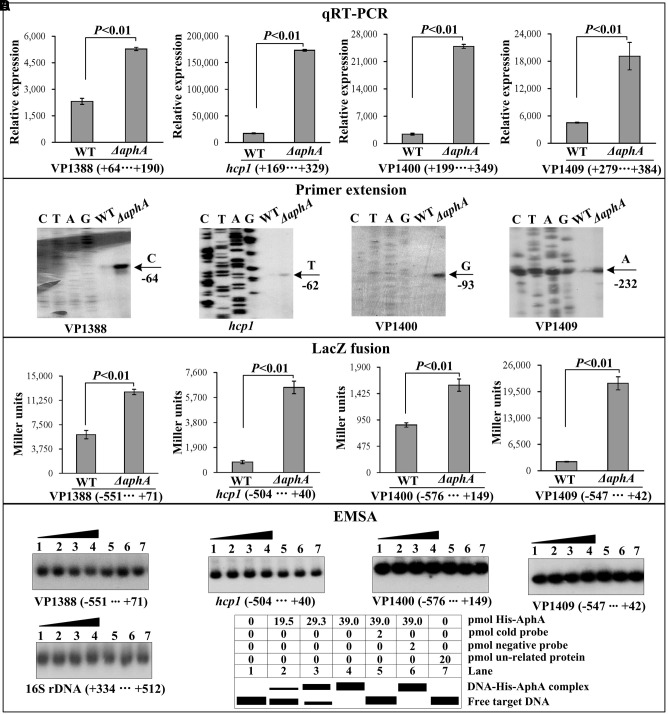
Regulation of T6SS1 genes by AphA. The negative and positive numbers represent the nucleotide position upstream and downstream of each target gene, respectively. Lanes C, T, A and G represent the Sanger sequencing reactions. **(A)** qRT-PCR. The relative mRNA level of each target gene was compared between Δ*aphA* and WT. **(B)** Primer extension. An oligonucleotide primer was designed to be complementary to the RNA transcript of each target gene. The primer extension products were analyzed with an 8 M urea -6% acrylamide sequencing gel. The transcription start sites were indicated by the arrow with nucleotide and position. **(C)** LacZ fusion assay. The entire promoter-proximal region of each target gene was cloned into pHRP309, and then transformed into WT or Δ*aphA* to determine the β-galactosidase activity (miller units) in cellular extracts. **(D)** EMSA. The entire promoter-proximal region of each target gene was incubated with increasing amounts of purified His-AphA protein, and then subjected to 6% (w/v) polyacrylamide gel electrophoresis. Shown below the binding was the schematic representation of the EMSA design.

As determined by the qRT-PCR assay (**Figure 2A**), the mRNA level of all the four genes, i.e., VP1388, *hcp1*, VP1400, and VP1409, was enhanced in Δ*aphA* relative to WT. The primer extension assay (**Figure 2B**) further indicated that the mRNA levels of the four genes were enhanced in Δ*aphA* relative to WT. The recombinant *lacZ* fusion plasmid that contains the indicated promoter-proximal region and promoterless *lacZ* gene was transformed into Δ*aphA* and WT, respectively, to test the action of AphA on the promoter activity of the above four genes. The results disclosed a significantly enhanced promoter activity of each of the four genes in Δ*aphA* relative to WT (**Figure 2C**). The promoter DNA regions of the above four genes were amplified, purified, radioactively labeled, and then subjected to EMSA with the purified His-AphA (**Figure 2D**). The results showed that His-AphA was unable to bind to the upstream DNA fragment of each target promoters, these were consistent with the predict results (**Table 2**). Taken together, AphA appears to negatively regulate the transcription of VP1388-1390, VP1393-1406, VP1400-1406, and VP1409-1407 in an indirect manner.

### Negative Regulation of T6SS1 by OpaR

**FIGURE 3:**
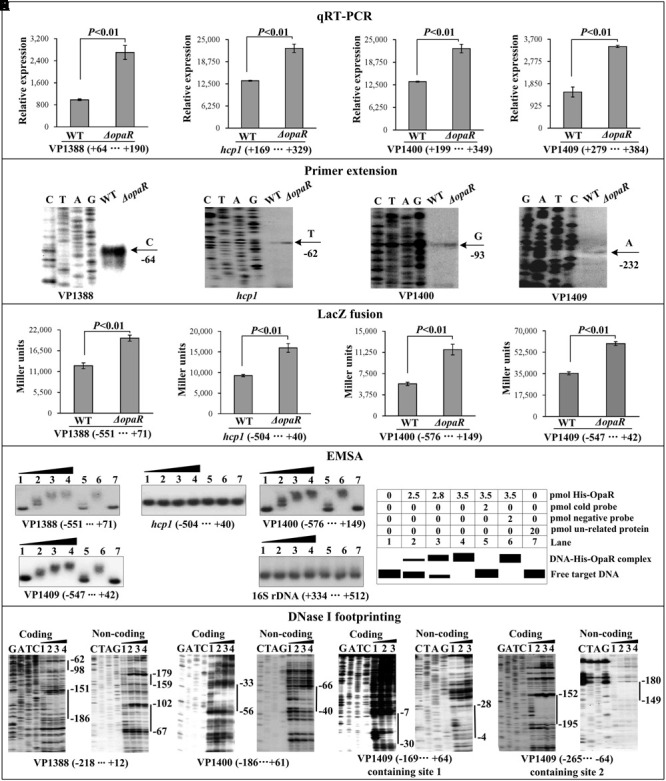
Regulation of T6SS1 genes by OpaR. The qRT-PCR **(A)**, primer extension **(B)**, LacZ fusion **(C)**, and EMSA **(D)** were done as **Figure 2**. **(E)** DNase footprinting assay. Labeled coding or non-coding DNA probes were incubated with increasing amounts of purified His-OpaR, and subjected to DNase I footprinting assay. The footprint regions were indicated with vertical bars.

The qRT-PCR and primer extension assays indicated that the transcription of all the four target genes VP1388, *hcp1*, VP1400, and VP1409 increased in Δ*opaR* relative to WT (**Figures 3A,B**). The *lacZ* fusion results showed that the promoter activity of each of the four operons in Δ*opaR* was much higher relative to that in WT (**Figure 3C**). The EMSA results showed that His-OpaR was able to bind to the upstream DNA fragment of VP1388, VP1400, and VP1409 in a dose dependent manner, but a negative EMSA result was observed for *hcp1* (**Figure 3D**). His-OpaR at all amounts used could not bind to the 16S rDNA fragment as the negative control (**Figure 3D**). As further determined by DNA footprinting (**Figure 3E**), His-OpaR protected two different DNA regions upstream of VP1388 and VP1409 against DNase I digestion that were considered as the OpaR sites, while only a single OpaR site was detected for VP1400. Taken together, OpaR represses the transcription of VP1388-1390, VP1400-1406, and VP1409-1407 in a direct manner, but it appears to negatively regulate the transcription of VP1393-1406 in an indirect manner.

### Negative Regulation of T6SS1by ToxR

**FIGURE 4:**
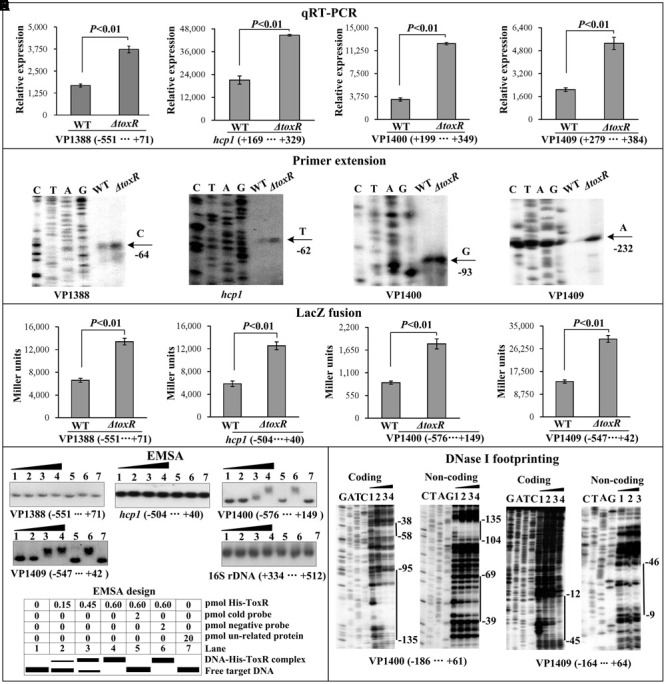
Regulation of T6SS1 genes by ToxR. The qRT-PCR **(A)**, primer extension **(B)**, LacZ fusion **(C)**, and EMSA **(D)** were done as **Figure 2**. The DNase footprinting assay **(E)** was done as **Figure 3**.

As determined by the qRT-PCR assay (**Figure 4A**), the mRNA level of each target gene was greatly increased in Δ*toxR* relative to WT. The primer extension assay detected a single ToxR-repressed transcription start site for each of the four target operons (**Figure 4B**). The *lacZ* fusion results showed that the promoter activity of each of the four operons in Δ*toxR* was significantly enhanced than that in WT (**Figure 4C**). The EMSA results showed that His-ToxR was able to bind to the upstream DNA fragment of VP1400 and VP1409 in a dose dependent manner, but negative EMSA results were observed for VP1388 and *hcp1* (**Figure 4D**). As further determined by DNA footprinting (**Figure 4E**), His-ToxR protected one or more DNA regions upstream of VP1409 or VP1400 against DNase I digestion that were considered as the ToxR sites. Taken together, ToxR represses the transcription of VP1400-1406 and VP1409-1407 in a direct manner, but it appears to negatively regulate the transcription of VP1388-1390 and VP1393-1406 in an indirect manner.

### Promoter Structure of Indicated Target Genes

**FIGURE 5:**
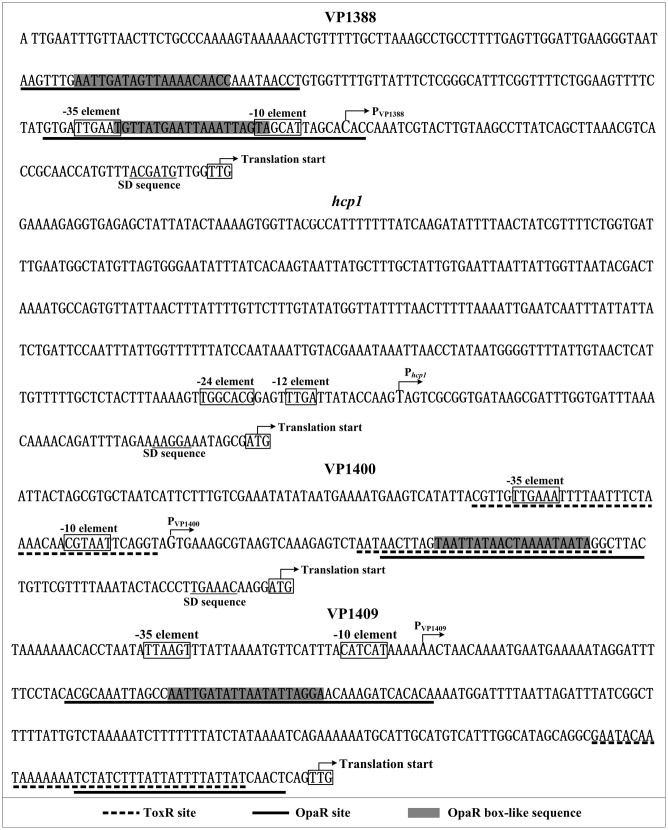
Structural organization of target promoters. The DNA sequence was derived from *V. parahaemolyticus* RIMD 221063. The transcription start sites were indicated by bent arrows. Shine-Dalgarno (SD) box and –10/–35 elements were enclosed in boxes. The OpaR sites were underlined with solid lines, while the ToxR sites were underlined with dotted lines.

Collection of data of translation/transcription start sites, promoter -10 and -35 (or -12 and -24) elements, AphA/OpaR/ToxR sites, AphA/OpaR/ToxR box-like sequences, Shine-Dalgarno (SD) sequences (ribosomal binding sites) enabled us to depict the organization of VP1388, *hcp1*, VP1400 and VP1409 promoters characterized herein (**Figure 5**).

## Discussion

**FIGURE 6:**
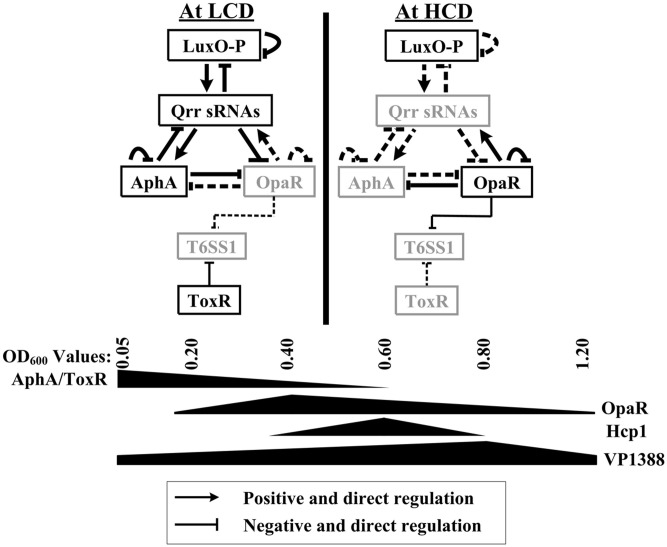
Regulation model. The regulatory behaviors between LuxO, Qrr sRNAs, AphA, and OpaR were described previously in *V. parahaemolyticus* and closely related *V. harveyi* (Henke and Bassler, 2004; Tu et al., 2010; Rutherford et al., 2011; Sun et al., 2012; Zhang et al., 2012). AphA and OpaR are the two master QS regulators that operate at low cell density (LCD) and high cell density (HCD), respectively. The regulatory protein ToxR, which is expressed at LCD, coordinates with QS to repress T6SS1 expression from LCD to physiological HCD, resulting in high expression of T6SS1 at the mid-logarithmic growth phase.

Many bacterial genomes harbor more than one T6SS loci that are involved in different cellular functions (Kapitein and Mogk, 2013), suggesting the T6SS loci would be differently regulated by various factors including regulatory proteins. In the present report, we provided evidence that the T6SS1 loci in *V. parahaemolyticus* is under the negative control of both QS and the virulence regulator ToxR (**Figure 6**). At HCD (OD_600_ = 0.4–0.6), OpaR binds to the three promoters of VP1388-1390, VP1400-1406, and VP1409-1407 to repress their transcription, but it appears to negatively regulate VP1393-1406 transcription in an indirect manner. At LCD (OD_600_ = 0.05–0.2), AphA negatively regulated the above four T6SS1 operons in an indirect manner; while ToxR binds to the promoters of VP1400-1406 and VP1409-1407 to inhibit their transcription, but it presents an indirect interaction with VP1388-1390 and VP1393-1406 promoters. The highest transcription of T6SS1 genes occurred at an OD_600_ value of 0.6 to 0.8 due to the tight regulation of ToxR and QS, suggesting T6SS1 may function at the later stages of HCD.

The QS-dependent expression of T6SSs has arisen in literature. Ishikawa et al. (2009) found that expression of Hcp was strictly and positively regulated by the QS regulator HapR in *V. cholerae* O1 strain A1552. Zheng et al. (2010) further showed that *V. cholerae* O1 strain C6707 HapR directly binds to the promoter regions of the T6SS genes *hcp1* and *hcp2* to induce their expression. The T6SS in *Aeromonas hydrophila* is also under positive control of AhyR, an *N*-acyl homoserine lactone (s) -mediated quorum regulator, the *ahyR* and *ahy*I double knockout mutant is unable to secrete T6SS-associated effectors (Khajanchi et al., 2009). However, not all bacteria T6SSs are positively regulated by the HCD QS regulators. In *Pseudomonas aeruginosa*, the H1-T6SS is suppressed at HCD by QS regulators, whereas both H2-T6SS and H3-T6SS loci are up-regulated by QS regulators during the growth phase transition (Lesic et al., 2009; Sana et al., 2012). In the fish pathogen *V. alginolyticus*, the expression of T6SS1 gene *hcp1* is positively and negatively regulated by QS regulators LuxO and LuxR, respectively (Sheng et al., 2012). Gode-Potratz and McCarter (2011) showed that OpaR strongly and oppositely regulates two T6SSs in *V. parahaemolyticus*. OpaR binds to the promoter regions of T6SS2 genes to activate their transcription, while AphA was shown to negatively regulate their transcription in an indirect manner (Wang et al., 2013). However, the detailed mechanisms of QS-dependent expression of *V. parahaemolyticus* T6SS1 are still obscure. The data presented here demonstrated that OpaR directly represses the transcription of T6SS1 genes VP1388-1390, VP1400-1406, and VP1409-1407, while AphA appears to repress their transcription in an indirect manner. More importantly, ToxR is also directly involved in regulating T6SS1 genes expression. Although ToxR-dependent expression of the major virulence determinants TDH and T3SS has been elucidated previously (Lin et al., 1993; Whitaker et al., 2012; Hubbard et al., 2016; Osei-Adjei et al., 2017), here we reported the regulation mechanisms of T6SS by ToxR in *V. parahaemolyticus*. However, Salomon et al. (2014) found that AphA and ToxR have a mild positive effect on Hcp1 expression under marine-like conditions (Luria–Bertani broth containing 3% sodium chloride) at warm temperature (30°C) in the absence of surface-sensing activation, while have no apparent effect on Hcp1 expression and secretion or on T6SS1 anti-bacterial activity under the surface-sensing activation, when used the POR1 (*V. parahaemolyticus* RIMD 2210633 Δ*tdhAS*) as parental strain (Salomon et al., 2014). Although our observations are only at the transcriptional level, it appears that the regulation of T6SS1 by QS and ToxR depends on the bacterial growth conditions and genetic background.

The highest transcript levels of *hcp1* and VP1388 occurred at an OD_600_ value of 0.6 and 0.8, respectively, due to the QS regulation (**Figure 1**), suggesting *V. parahaemolyticus* T6SS1 functions only during the mid-logarithmic growth phase. Similar observations have been reported in other species, including *Yersinia pseudotuberculosis* (Zhang et al., 2011), *V. cholerae* (Ishikawa et al., 2009), *V. alginolyticus* (Sheng et al., 2012), *V. anguillarum* (Tang et al., 2016), and *V. fluvialis* (Huang et al., 2017). Notably, the highest expression levels of AphA and ToxR occurred at an OD_600_ value of 0.05 to 0.2, whereas that of OpaR occurred at 0.4–0.6. These results indicated that low production of AphA, OpaR and ToxR couldn’t effectively inhibit the T6SS1 expression, or there may be another regulatory factor that can activate the transcription of T6SS1 genes when the bacteria strain was grown in M broth. Cell density-dependent transcription of ToxR has been observed in *V. cholerae* (Xu et al., 2010), suggesting a possible and conservative connection between ToxR transcription and QS in pathogenic *vibrios*. This connection would be beneficial for the tight regulation of the fitness and virulence during growth and pathogenesis. However, the mechanism of ToxR integration into the QS signal transduction needs to be further investigated.

Based on the primer extension and DNase I footprinting data, we reconstructed the structural organization of each indicated promoters. As shown in **Figure 5**, two OpaR sites were detected for VP1388-1390, and one of which overlaps the core promoter -10 and -35 and the transcription start site; the OpaR sites for VP1400-1406 and VP1409-1407 are located downstream of the transcription start site. Thus, the binding of OpaR would block the entry or elongation of the RNA polymerase to repress the transcription of the target genes. Notably, each of the OpaR sites contains the OpaR box-like sequence except for the site next to the translation start of VP1409-1407, suggesting the binding sites identified by bioinformatics is not a guarantee that a protein binds to a region or not, it is still possible that other sites may exist. ToxR bound one/two sites within the upstream region of VP1409-1407/VP1400-1406, and (one of) the binding site overlaps with the corresponding OpaR site, indicating the repression mechanisms by ToxR would be similar to that of by OpaR. However, we didn’t detect the ToxR box-like sequence within all the ToxR sites, suggesting the consensus sequence of TNAAA-N5-TNAAA identified in *V. cholerae* (Goss et al., 2013) does not have universal applicability in other *vibrios*.

Collectively, this work reported that ToxR coordinates with QS regulators AphA and OpaR to repress T6SS1 in *V. parahaemolyticus* strain RIMD2210633, leading to the highest transcription of T6SS1 genes occurred at the mid-logarithmic growth phase (i.e., the later stages of HCD) when grown the bacteria cells in M broth. Thus, T6SS1 genes are assigned as the members of QS and ToxR regulons in *V. parahaemolyticus*.

## Author Contributions

DZ and XH conceived the study and designed experimental procedures. YiqZ, HG, GO-A, YinZ, WY, HY, and ZY performed the experiments and carried out data analysis. YiqZ, GO-A, DZ, and XH wrote the paper.

## Conflict of Interest Statement

The authors declare that the research was conducted in the absence of any commercial or financial relationships that could be construed as a potential conflict of interest.

## Footnotes

1 http://embnet.ccg.unam.mx/rsat/

## Supplementary Material

FIGURE S1 Organization of the T6SS gene clusters. Genes that were investigated in this study are presented as boldface word. Sequences between the arrows are the putative promoter regions that were tested in the present work.

FIGURE S2 Non-polar deletion of Δ*aphA*, Δ*opaR*, and Δ*toxR*. *V. parahaemolyticus* cells were grown in M broth containing 5 μg/ml chloramphenicol and 0.1% arabinose. The primer extension **(A)** and qRT-PCR **(B)** assays were subsequently employed to determine the relative mRNA levels of *hcp1* in WT/pBDA33, Δ*aphA*/pBDA33, Δ*aphA/*pBDA33-*aphA*, Δ*opaR*/pBDA33, Δ*opaR/*pBDA33- *opaR*, Δ*toxR*/pBDA33, and Δ*toxR/*pBDA33-*toxR*. The *hcp1* mRNA level was significantly activated in Δ*aphA*/pBDA33 relative to either WT/pBDA33 or Δ*aphA/*pBDA33-*aphA*, and the similar results were also observed in Δ*opaR*/pBDA33 relative to WT/pBDA33 and Δ*opaR/*pBDA33-*opaR* or Δ*toxR*/pBDA33 relative to WT/pBDA33 and Δ*toxR/*pBDA33-*toxR.* These results confirmed that the Δ*aphA*, Δ*opaR*, and Δ*toxR* strains were non-polar.
