# Corrigendum: Transcriptional Regulation of the Type VI Secretion System 1 Genes by Quorum Sensing and ToxR in *Vibrio parahaemolyticus*

**DOI:** 10.3389/fmicb.2020.616153

**Published:** 2020-11-13

**Authors:** Yiquan Zhang, He Gao, George Osei-Adjei, Ying Zhang, Wenhui Yang, Huiying Yang, Zhe Yin, Xinxiang Huang, Dongsheng Zhou

**Affiliations:** ^1^School of Medicine, Jiangsu University, Zhenjiang, China; ^2^State Key Laboratory for Infectious Disease Prevention and Control, National Institute for Communicable Disease Control and Prevention, Chinese Centre for Disease Control and Prevention, Beijing, China; ^3^Department of Biosafety, State Key Laboratory of Pathogen and Biosecurity, Beijing Institute of Microbiology and Epidemiology, Beijing, China

**Keywords:** *Vibrio parahaemolyticus*, T6SS1, quorum sensing, AphA, OpaR, ToxR

In the original article, there was an error. The bacterial growth condition was mistakenly described as in Difco marine broth 2216 (BD Biosciences) at 37°C with shaking at 200 rpm.

A correction has been made to the section Materials and Methods, Bacterial Growth:

“*Vibrio parahaemolyticus* strains were cultured in Difco marine broth 2216 (BD Biosciences) at 30°C with shaking at 200 rpm.”

The authors apologize for this error and state that this does not change the scientific conclusions of the article in any way. The original article has been updated.

