# Supplementary figures and images for: Transcriptional Regulation of the Type VI Secretion System 1 Genes by Quorum Sensing and ToxR in Vibrio parahaemolyticus
Source: Front Microbiol. 2017 Oct 16;8:2005. doi: 10.3389/fmicb.2017.02005 (PMC5650642; doi:10.3389/fmicb.2017.02005)

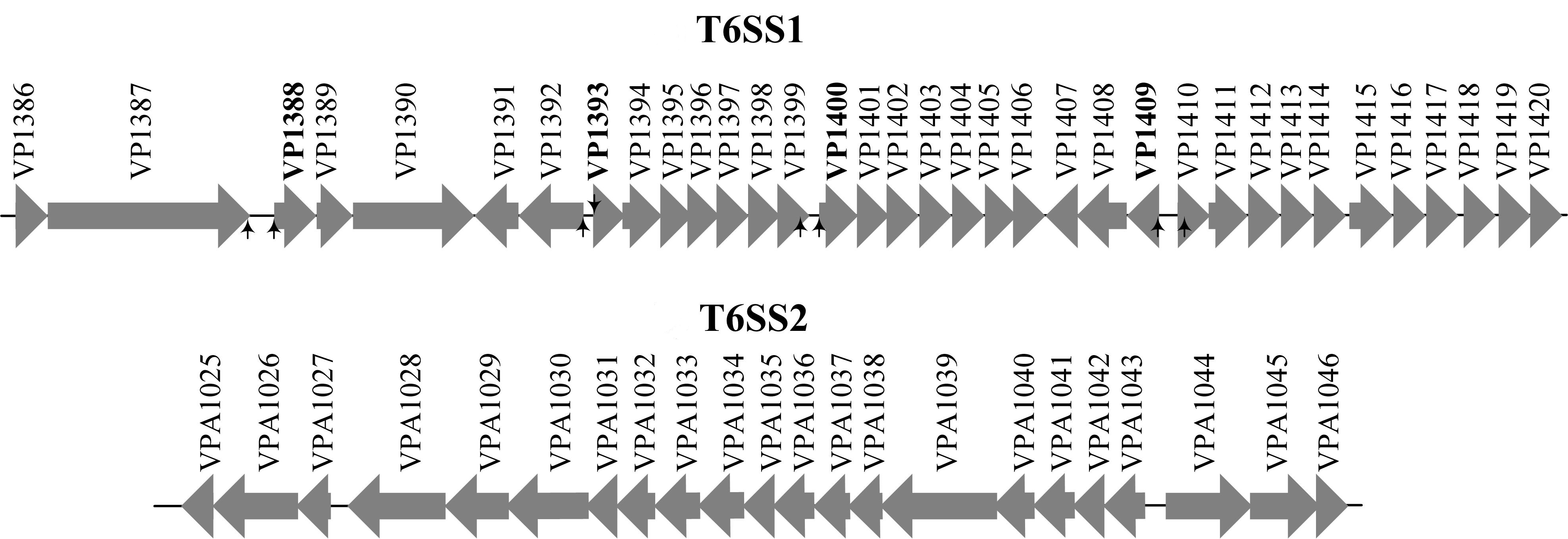

Supplement: FIGURE S1 — Organization of the T6SS gene clusters. Genes that were investigated in this study are presented as boldface word. Sequences between the arrows are the putative promoter regions that were tested in the present work. [file Image_1.jpg]

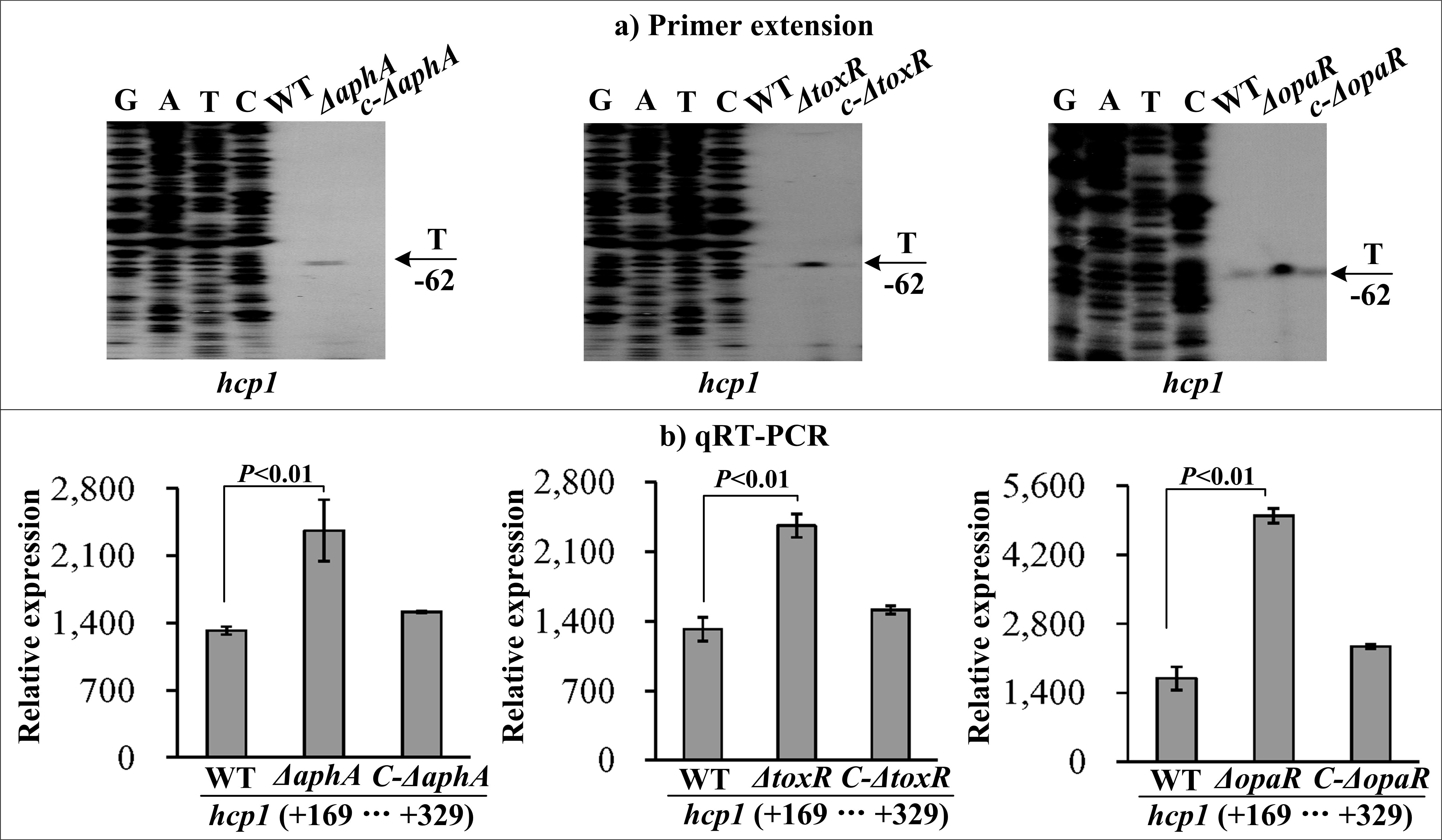

Supplement: FIGURE S2 — Non-polar deletion of ΔaphA, ΔopaR, and ΔtoxR. V. parahaemolyticus cells were grown in M broth containing 5 μg/ml chloramphenicol and 0.1% arabinose. The primer extension (A) and qRT-PCR (B) assays were subsequently employed to determine the relative mRNA levels of hcp1 in WT/pBDA33, ΔaphA/pBDA33, ΔaphA/pBDA33-aphA, ΔopaR/pBDA33, ΔopaR/pBDA33- opaR, ΔtoxR/pBDA33, and ΔtoxR/pBDA33-toxR. The hcp1 mRNA level was significantly activated in ΔaphA/pBDA33 relative to either WT/pBDA33 or ΔaphA/pBDA33-aphA, and the similar results were also observed in ΔopaR/pBDA33 relative to WT/pBDA33 and ΔopaR/pBDA33-opaR or ΔtoxR/pBDA33 relative to WT/pBDA33 and ΔtoxR/pBDA33-toxR. These results confirmed that the ΔaphA, ΔopaR, and ΔtoxR strains were non-polar. [file Image_2.jpeg]
